# Electrochemical Sensing of Estradiol Using an Fe_3_O_4_/fMWCNT-Modified Carbon Paste Electrode

**DOI:** 10.3390/s26216917

**Published:** 2026-10-31

**Authors:** Ana Đurović, Zorica Stojanović, Sanja Panić, Marija Milanović, Snežana Kravić, Lukáš Richtera

**Affiliations:** 1 Faculty of Technology Novi Sad, University of Novi Sad, Bul. cara Lazara 1, 21102 Novi Sad, Serbia; zorica.stojanovic@uns.ac.rs (Z.S.); sanjar@tf.uns.ac.rs (S.P.); majam@uns.ac.rs (M.M.); sne@uns.ac.rs (S.K.); 2 Department of Chemistry and Biochemistry, Mendel University in Brno, Zemědělská 1, 613 00 Brno, Czech Republic; lukas.richtera@mendelu.cz

**Keywords:** estradiol, carbon paste electrode, Fe_3_O_4_/fMWCNTs, square-wave voltammetry, pharmaceutical analysis

## Abstract

A sensitive and reproducible electroanalytical method was developed for the selective determination of estrogen hormone estradiol (EST) using a carbon paste electrode (CPE) modified with Fe_3_O_4_-functionalized multi-walled carbon nanotubes (Fe_3_O_4_/fMWCNTs-CPE). The Fe_3_O_4_/fMWCNT composite was synthesized via wet impregnation and characterized by field-emission scanning electron microscopy, energy-dispersive X-ray spectroscopy, and X-ray diffraction, while the sensor’s electrochemical and interfacial properties were assessed using cyclic voltammetry and electrochemical impedance spectroscopy. Electrochemical studies revealed an irreversible oxidation peak for EST, and square-wave voltammetry was optimized for its determination in 0.1 mol/L phosphate buffer (pH 5.0). Under optimal conditions, the sensor exhibited a linear dynamic range (up to 20 µM) with a detection limit of 21.5 nM, indicating high sensitivity. Moreover, it showed good selectivity in the presence of common interfering substances. The method was successfully applied for EST determination in one urine sample, one water sample, and one pharmaceutical tablet sample, achieving high recovery values (98.53–101.79%), with RSD values below 2.40%, requiring only sample dilution without additional pretreatment.

## 1. Introduction

Hormones are organic substances that regulate numerous biological functions, including growth, metabolism, tissue differentiation, and the development of the sexual and immune systems. One of the main female sex hormones are estrogens, with β-estradiol (hereafter referred to as estradiol, or EST) as one of the most potent members [1]. EST is synthesized mainly by the ovaries and adrenal glands, and plays a crucial role in reproduction, bone health, metabolism, and lipid storage [1]. It also modulates brain function, offering neuroprotection and aiding recovery from injury and degeneration [1]. As a key estrogen, EST is widely utilized in hormone therapy and fertility treatments, both in its natural and synthetic forms.

Humans and animals excrete significant amounts of natural and synthetic hormones, with livestock contributing the most [2]. These hormones enter the aquatic environment through sewage and animal waste [3], raising concerns about long-term effects on wildlife and human health, including reproductive disruptions, immune dysfunction, developmental issues, and increased tumor risk [4,5,6]. Due to the detection of residues in water, the European Union has proposed monitoring EST as a contaminant [3,7,8,9,10].

Various analytical approaches, including liquid chromatography–mass spectrometry [11,12,13], high-performance liquid chromatography [14,15,16,17,18,19], gas chromatography [20], spectrophotometry [19], and immunoassays [21,22,23], have been developed for EST detection. However, these methods often require extensive sample preparation and costly equipment. Electroanalytical methods offer a promising alternative, enabling direct, sensitive, and selective EST detection, with reduced costs and minimal sample preparation.

The design of the working electrode plays a crucial role in the performance of electroanalytical methods. Carbon paste electrodes (CPEs) have gained widespread use in sensing applications due to their advantageous properties, such as ease of preparation, simple surface modification and renewal, good mechanical resistance, and stability [24]. To enhance electrode performance, researchers typically employ a modification process, which is particularly straightforward in the case of CPEs, as it mostly involves incorporating specific materials during paste preparation [25,26]. Among these, multi-walled carbon nanotubes (MWCNTs) are particularly valued for their high surface area, low charge-transfer resistance, electrocatalytic activity, reduced fouling, chemical stability, and excellent mechanical strength [27,28,29]. Magnetite (Fe_3_O_4_) is another material of interest in electrochemistry, due to its unique electrical and magnetic properties. It has been widely applied in areas such as energy storage, electrocatalysis, electrochemical sensing, water treatment, and drug delivery [30,31,32,33].

This study describes the synthesis of functionalized multi-walled carbon nanotubes (fMWCNTs) with immobilized magnetite, forming a hybrid electrocatalyst (Fe_3_O_4_/fMWCNTs). This composite material was used to fabricate a modified CPE (Fe_3_O_4_/fMWCNTs-CPE), which was then evaluated for the voltammetric determination of EST. While previous studies have explored modified CPEs for EST detection [34,35,36,37], including those incorporating magnetite [38] and magnetic molecularly imprinted polymers [39], this approach differs by utilizing a novel combination of magnetite with fMWCNTs and a CPE, aiming to improve conductivity and electrocatalytic efficiency. The unique modification is designed to enhance sensitivity and selectivity for EST detection, distinguishing it from the existing CPE-EST sensors. The synthesized modifier was thoroughly characterized, and its interfacial properties were examined electrochemically. Following optimization and validation, the developed method was preliminarily evaluated for EST determination in various real samples using square-wave voltammetry (SWV).

## 2. Materials and Methods

### 2.1. Reagents and Solutions

EST (≥98% purity) was procured from Sigma-Aldrich (St. Louis, MO, USA). To prepare a 1 mM EST standard solution, the precise amount of the solid substance was initially dissolved in ethanol (10%, *v*/*v*) and subsequently diluted in a 0.1 M NaOH solution. Ethanol (96%) was obtained from Fisher Scientific (Waltham, MA, USA), and NaOH from Lachner (Brno, Czech Republic).

A 0.1 M phosphate buffer at a pH of 5.0 was prepared by mixing NaH_2_PO_4_ and Na_2_HPO_4_, and the pH was adjusted with 8.25 M NaOH. The expanded graphite for the working electrode preparation was obtained from Graphyte Tyn Ltd. (Tyn nad Vltavou, Czech Republic), and paraffin oil was sourced from Sigma-Aldrich (St. Louis, MO, USA). K_4_[Fe(CN)_6_]·3H_2_O, K_3_[Fe(CN)_6_], and KCl were purchased from Merck (Darmstadt, Germany). Double-distilled water was utilized throughout the analysis. All reagents used were of analytical reagent grade and were employed without further purification.

### 2.2. Instrumentation

The synthesized Fe_3_O_4_/fMWCNT composite was characterized by field emission (FE) scanning electron microscopy (SEM) (JEOL JSM-6460LV on 25 kV) with energy-dispersive spectroscopy (EDX) (Thermo Fisher Scientific Apreo 2C), while X-ray powder diffraction analysis (XRD) was conducted on a Rigaku MiniFlex 600 instrument using CuKα radiation with a wavelength of λ = 0.15406 nm.

Electrochemical measurements, including cyclic voltammetry (CV), SWV, and electrochemical impedance spectroscopy (EIS), were conducted using a PalmSens 4 potentiostat (PalmSens BV, GA Houten, The Netherlands) under the control of PSTrace software (version 5.9). The experiments were conducted within a three-electrode cell setup, wherein a platinum wire served as the counter electrode, Ag/AgCl (KCl, 3.5 M) was employed as the reference electrode, and Fe_3_O_4_/fMWCNTs-CPE, fMWCNTs-CPE, and bare CPE functioned as the working electrodes. pH adjustments were made using a pH meter HI-221 (Hanna Instruments, Woonsocket, RI, USA), equipped with a combined glass electrode P11 (Sentek, Springwood, Essex, UK). All measurements were carried out at room temperature (25 ± 1 °C).

### 2.3. Preparation of the Modified CPE

#### 2.3.1. Preparation of fMWCNTs

MWCNTs were synthesized using the catalytic chemical vapor deposition technique in a custom-made reactor, following a previously described method [40]. During synthesis, an ethylene–nitrogen mixture (1:1) flowed for 1 h at 700 °C over an in situ reduced 5% Fe-Co/Al_2_O_3_ catalyst [41]. To eliminate the Al_2_O_3_ catalyst support, the material was refluxed in a 6 M NaOH solution at its boiling point. After thorough washing and drying, the remaining catalyst metal particles were removed by refluxing the sample in concentrated HCl for 6 h. Oxygen-containing functional groups were introduced to the surface of the MWCNTs by treating them in boiling concentrated HNO_3_ for 8 h, producing fMWCNTs.

#### 2.3.2. Preparation of Fe_3_O_4_/fMWCNT Composite

The washed and dried tubes were utilized as a support for the preparation of the Fe_3_O_4_/fMWCNT composite by wet impregnation at room temperature. Briefly, 9.5 g of fMWCNTs was combined with 100 mL of an aqueous solution containing 3.62 g of Fe(NO_3_)_3_·9H_2_O (0.0895 M; Centrohem, Stara Pazova, Serbia), corresponding to a nominal Fe loading of 5 wt.%, calculated relative to the combined mass of Fe and fMWCNTs. The suspension was ultrasonically treated for 3 h, then mechanically stirred for 24 h. After drying, the resulting powder was thermally treated at 400 °C under an N_2_ flow of 110 mL min^−1^ for 3 h, then reduced at 400 °C under an H_2_ flow of 110 mL min^−1^ for 1 h to obtain the Fe_3_O_4_/fMWCNT composite.

#### 2.3.3. Preparation of CPE, fMWCNTs-CPE and Fe_3_O_4_/fMWCNTs-CPE

To prepare bare CPE, expanded graphite and paraffin oil were mixed in a 70/30 (*w*/*w*) ratio. The mixture was thoroughly blended for approximately 30 min using a mortar. The resulting paste was then packed into a Teflon tube with an inner diameter of 4 mm, and a copper wire was inserted to establish electrical contact. To achieve a smooth electrode surface, an excess of the paste was removed, and the electrode was polished on wet paper until a glossy finish was achieved, followed by rinsing with double-distilled water. After each set of experiments, a small portion of the carbon paste was extruded from the electrode body, and the electrode surface was renewed by polishing to obtain a fresh, flat surface.

Modified CPEs, including fMWCNTs-CPE and Fe_3_O_4_/fMWCNTs-CPE, were fabricated by incorporating the corresponding modifier into the carbon paste at 2.5% (*w*/*w*) relative to the mass of the solid phase. The subsequent steps involving homogenization, packing into a Teflon tube, and polishing were identical to those used for the bare CPE.

### 2.4. Measurement Procedure

Before conducting the voltammetric analysis, 10 mL of the supporting electrolyte was introduced into the voltammetric cell. The potential was then scanned in the anodic range. After obtaining the initial blank reading, precise volumes of EST standard solutions were added. The solutions were analyzed using SWV under optimized conditions: amplitude of 50 mV, frequency of 50 Hz, step potential of 7.5 mV, and equilibrium time of 0 s. Voltammetric measurements were generally performed in duplicate. Furthermore, CV was employed to investigate the oxidation mechanism of EST.

### 2.5. Electrochemical Characterization of Fe_3_O_4_/fMWCNTs-CPE

The electrochemical characterization of Fe_3_O_4_/fMWCNTs-CPE was performed using CV and EIS. For CV, the potential was cycled between −0.4 V and +0.9 V, employing a scan rate of 0.1 V/s and a step potential of 5 mV, in a 5 mM [Fe(CN)_6_]^3−/4−^ solution containing 0.1 M KCl. For EIS, a constant potential of 0.2 V was applied, with a frequency range from 0.1 Hz to 100,000 Hz, and a signal amplitude of 10 mV. The electrochemical characterization was performed in duplicate using the same electrode.

### 2.6. Sample Preparation

#### 2.6.1. Urine Sample

A urine sample from a healthy female volunteer was obtained immediately before the experiments. A 100 µL portion of freshly collected urine was introduced into the electrochemical cell containing 10 mL of the supporting electrolyte. The sample was spiked with an EST standard solution to achieve two concentration levels of EST: 2.5 µM and 5 µM. The hormone’s quantification was carried out using the standard addition method.

#### 2.6.2. Water Sample

A tap water sample was collected from our laboratory. A 2 mL portion of the freshly obtained water sample was transferred into the electrochemical cell, followed by adding 8 mL of the supporting electrolyte before the analysis. The diluted water sample was then spiked with an EST standard solution to achieve final EST concentrations of 1.25 µM, 2.5 µM, and 4.5 µM. The hormone content was assessed using the standard addition method.

#### 2.6.3. Pharmaceutical Formulation

A pharmaceutical dosage of EST in the form of Cyclo-Progynova film tablets (Bayer, Berlin, Germany) containing 2 mg of the active substance was procured from a local pharmacy in Novi Sad, Serbia. For quantitative analysis, three replicate determinations were performed using separate tablets from the same batch. Each tablet was crushed in a mortar using a pestle and quantitatively transferred into a 50 mL volumetric flask with 1 mL of ethanol and the supporting electrolyte. After sonication for 30 s, the solution volume was adjusted using the supporting electrolyte. An appropriate dilution was prepared in a separate volumetric flask to ensure the hormone concentration fell within the linear range for voltammetric analysis. Subsequently, 10 mL of the diluted sample was introduced into the electrochemical cell and analyzed utilizing the standard addition method.

## 3. Results and Discussion

### 3.1. FE-SEM/EDX Characterization of Fe_3_O_4_/fMWCNT Composite

**Figure 1:**
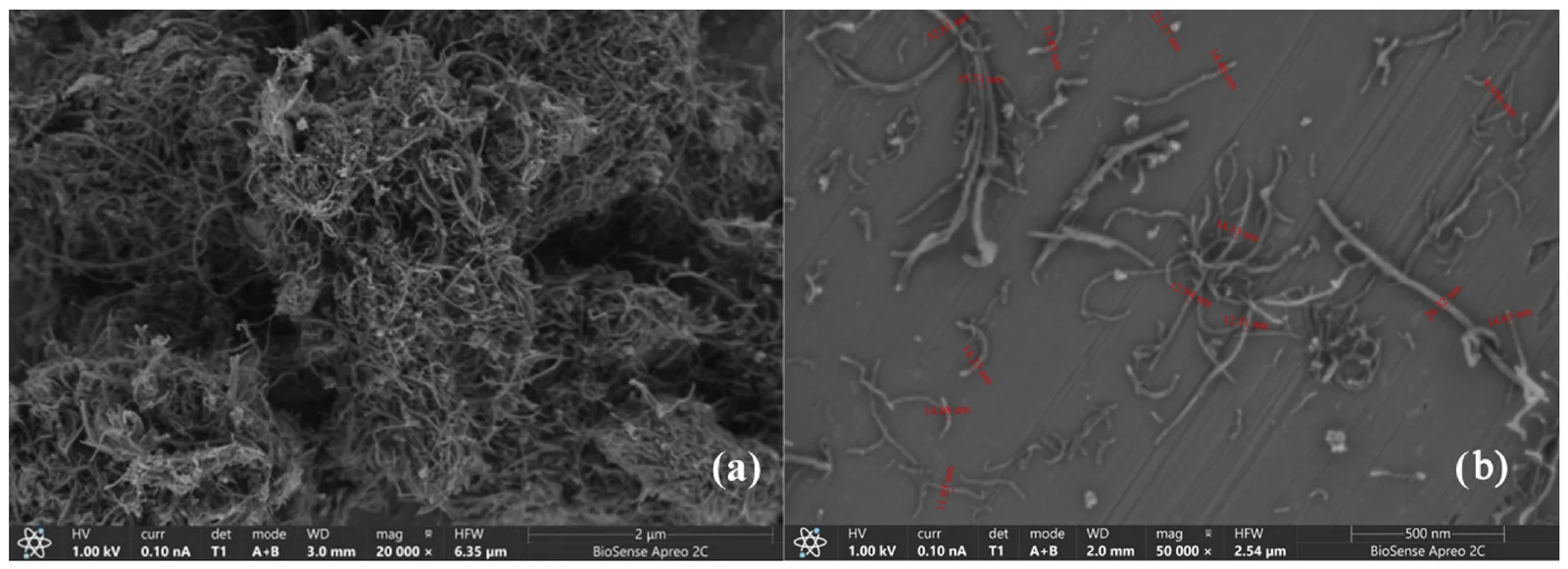
FE-SEM images of the Fe_3_O_4_/fMWCNT composite at magnifications of (**a**) 20,000× and (**b**) 50,000×.

The morphology of the synthesized Fe_3_O_4_/fMWCNT composite was analyzed using FE-SEM. Figure 1 presents images of the surface morphology of the composite at two different magnifications. The tubes exhibit highly interwoven and twisted structures, forming a three-dimensional network. Measurements at selected locations in Figure 1b indicate approximate external nanotube diameters ranging from 10 to 33 nm. Particles associated with the iron oxide phase are distributed along the nanotubes, predominantly as small aggregates.

**Figure 2:**
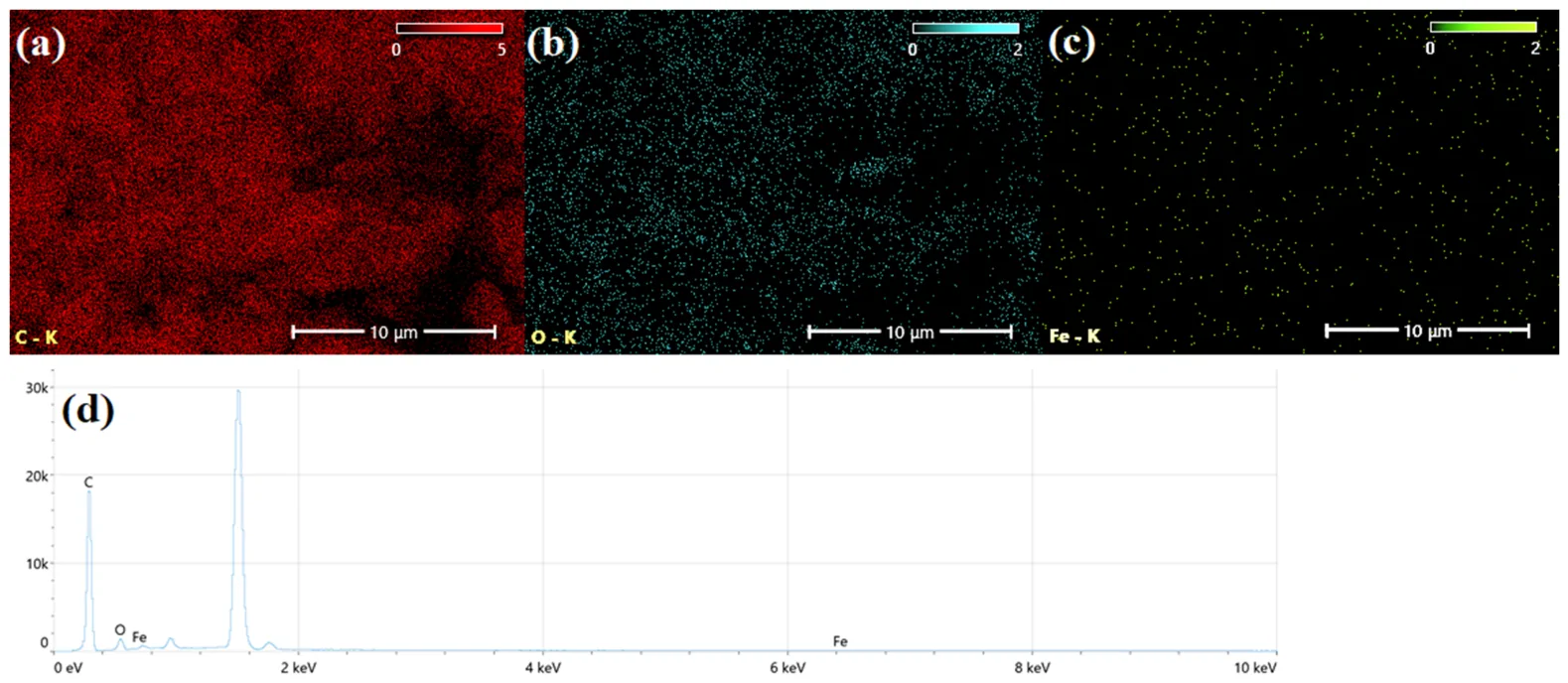
EDX element mapping for (**a**) carbon, (**b**) oxygen, (**c**) iron, and (**d**) EDX spectrum of the Fe_3_O_4_/fMWCNT composite.

EDX elemental mapping (Figure 2a–c) illustrates the spatial distribution of carbon, oxygen, and iron in the Fe_3_O_4_/fMWCNT composite. Iron signals are dispersed across the mapped area, supporting the presence of the iron-containing phase throughout the carbon-based material. The EDX spectrum (Figure 2d) confirms these elements, while quantitative EDX analysis gives carbon, oxygen, and iron contents of 77.3, 15.4, and 7.3 wt%, respectively.

### 3.2. XRD Analysis

**Figure 3:**
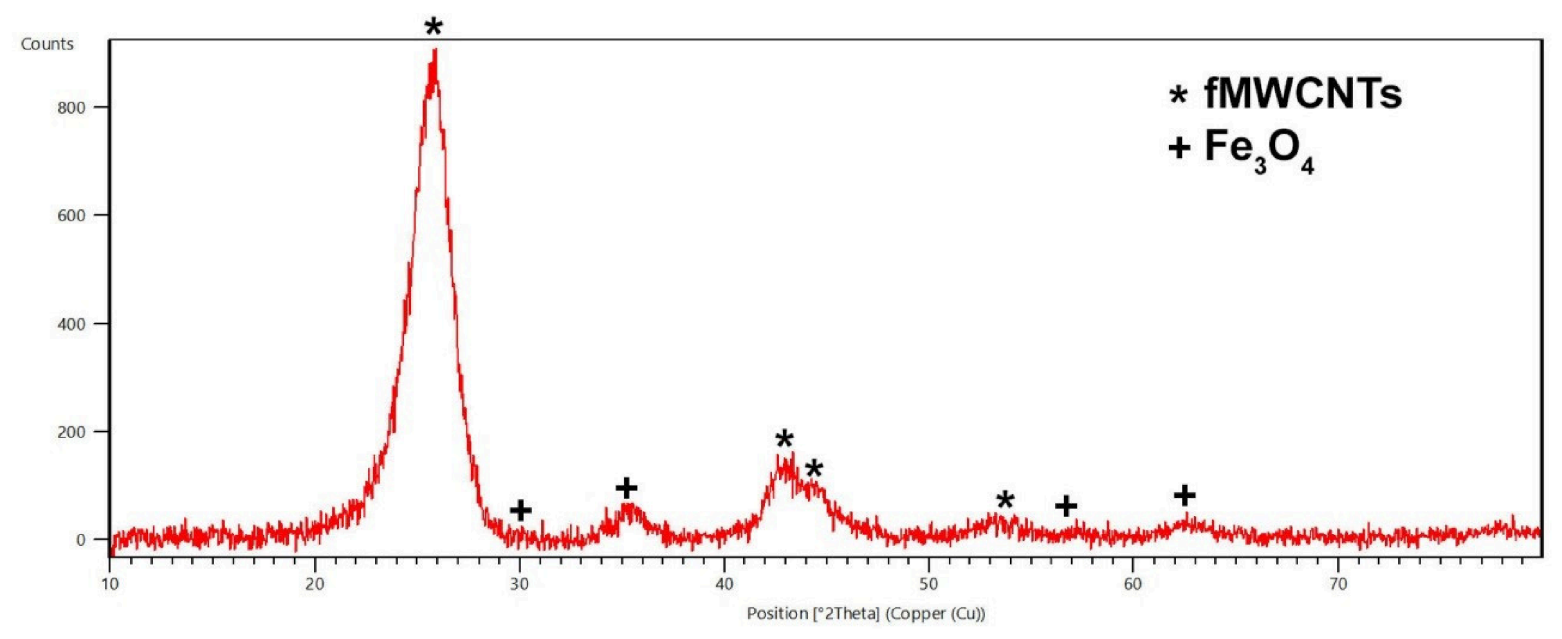
The red curve represents the experimental XRD pattern of the Fe_3_O_4_/fMWCNTs composite.

Figure 3 shows the X-ray diffraction pattern of the Fe_3_O_4_/fMWCNT composite. The observed reflections are consistent with a graphitic carbon phase and a spinel-type iron oxide phase assigned to Fe_3_O_4_. The reflection at 2Θ = 25.6° corresponds to the (002) planes of the graphitic structure of fMWCNTs. The reflections at 2θ = 30.1°, 35.4°, 56.8°, and 62.4° can be indexed to the (220), (311), (511), and (440) planes of the spinel iron oxide structure, respectively (Reference code: 96-900-9769). Using the (311) reflection at 2θ = 35.4° and Cu Kα radiation (λ = 0.15406 nm), the interplanar spacing was calculated as d_311_ ≈ 0.253 nm, corresponding to an estimated cubic lattice parameter of a ≈ 0.840 nm. The broadening of this reflection indicates nanocrystalline domains, with an apparent crystallite size of approximately 3.6 nm estimated using the Scherrer equation. The diffraction pattern supports a magnetite-type spinel structure, with a possible maghemite contribution due to the similarity of their characteristic reflections.

### 3.3. Electrochemical Behavior of EST

**Figure 4:**
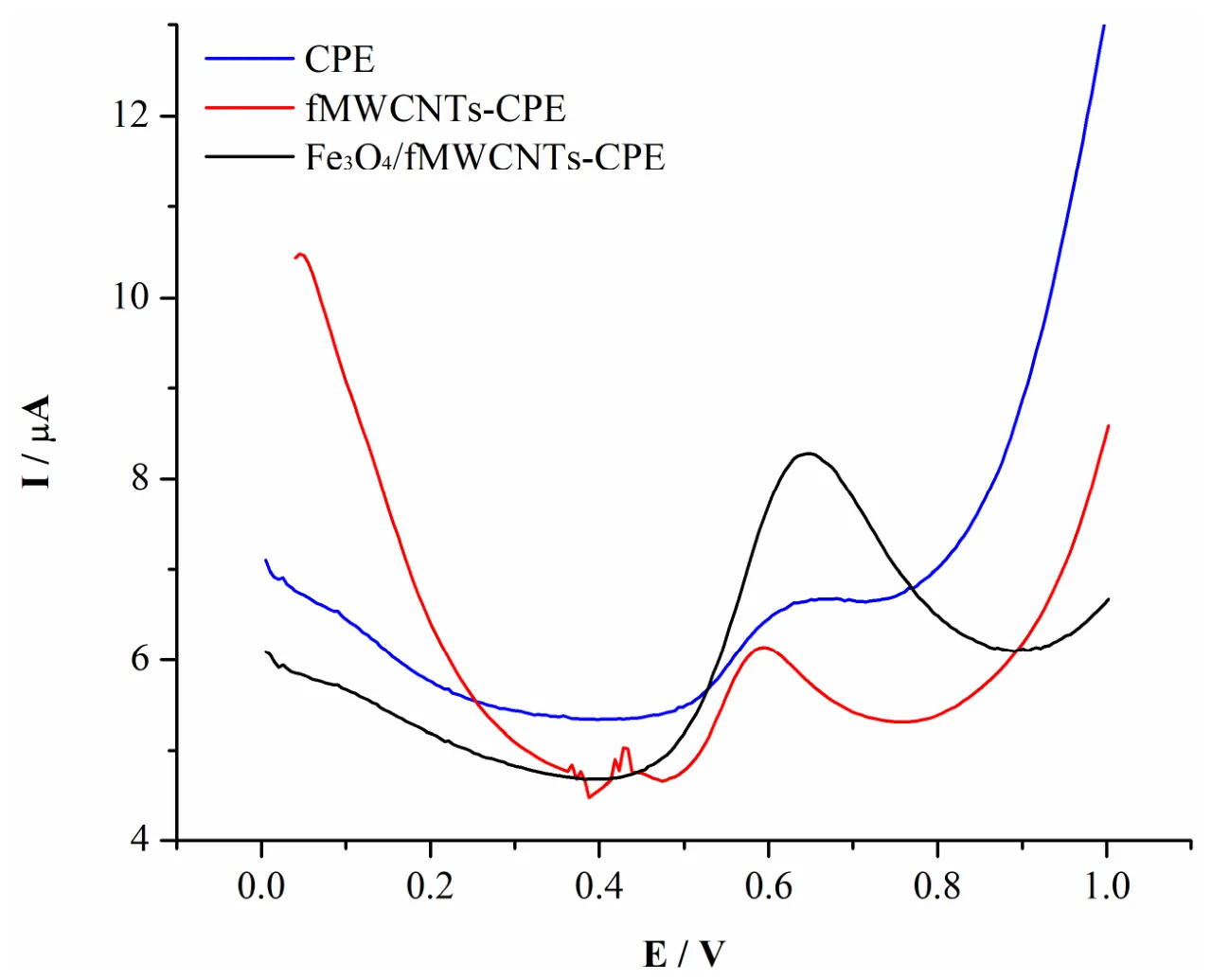
Comparative SWV analysis of different working electrodes (CPE, fMWCNTs-CPE, and Fe_3_O_4_/fMWCNTs-CPE) for electrochemical sensing of 20 μM EST in phosphate buffer solution (pH 5.0) (*t*_eq_ = 5 s, *ΔE* = 5 mV, *E*_sw_ = 50 mV, *f* = 50 Hz).

To evaluate the performance of different sensors for EST determination, various working electrodes were tested. The electrochemical behavior of 20 μM EST in phosphate buffer (pH 5.0) was investigated using SWV with CPE, fMWCNTs-CPE, and Fe_3_O_4_/fMWCNTs-CPE (Figure 4). The bare CPE produced a weak response and a broadened peak. Incorporating fMWCNTs into the paste led to an enhanced and better-defined oxidation peak, although some signal distortion, attributed to measurement-related noise, was observed under the applied experimental conditions. The Fe_3_O_4_/fMWCNTs-CPE exhibited the highest and best-defined oxidation peak among the tested electrodes, indicating an improved analytical response following the incorporation of the iron oxide phase into the fMWCNT-based modifier. However, the possible interaction between Fe_3_O_4_ and fMWCNTs could not be evaluated independently, since the properties of iron oxide prepared without the carbon support may differ from those of the oxide phase formed within the composite.

### 3.4. Effect of the Amount of Modifier

Optimizing the modifier content in the paste is crucial for enhancing the performance of the modified CPE. In this study, the effect of different Fe_3_O_4_/fMWCNT concentrations in CPE was evaluated using SWV in 0.1 M phosphate buffer (pH 5.0) containing 20 μM EST. For that purpose, paste formulations with 1%, 2.5%, 5%, and 10% (*w*/*w*, relative to solids content) of the modifier were prepared. The SWV results reveal a distinct trend: the anodic peak current increases with modifier concentration up to 2.5%, followed by a decline (Figure S1a,b). This decrease is likely due to an excessive modifier content hindering electron transfer, thereby reducing the analytical signal for EST. Consequently, the paste containing 2.5% Fe_3_O_4_/fMWCNTs was identified as optimal for fabrication, emphasizing the crucial role of modifier concentration in enhancing electrochemical performance for EST sensing.

### 3.5. Characterization of Fe_3_O_4_/fMWCNTs-CPE

**Figure 5:**
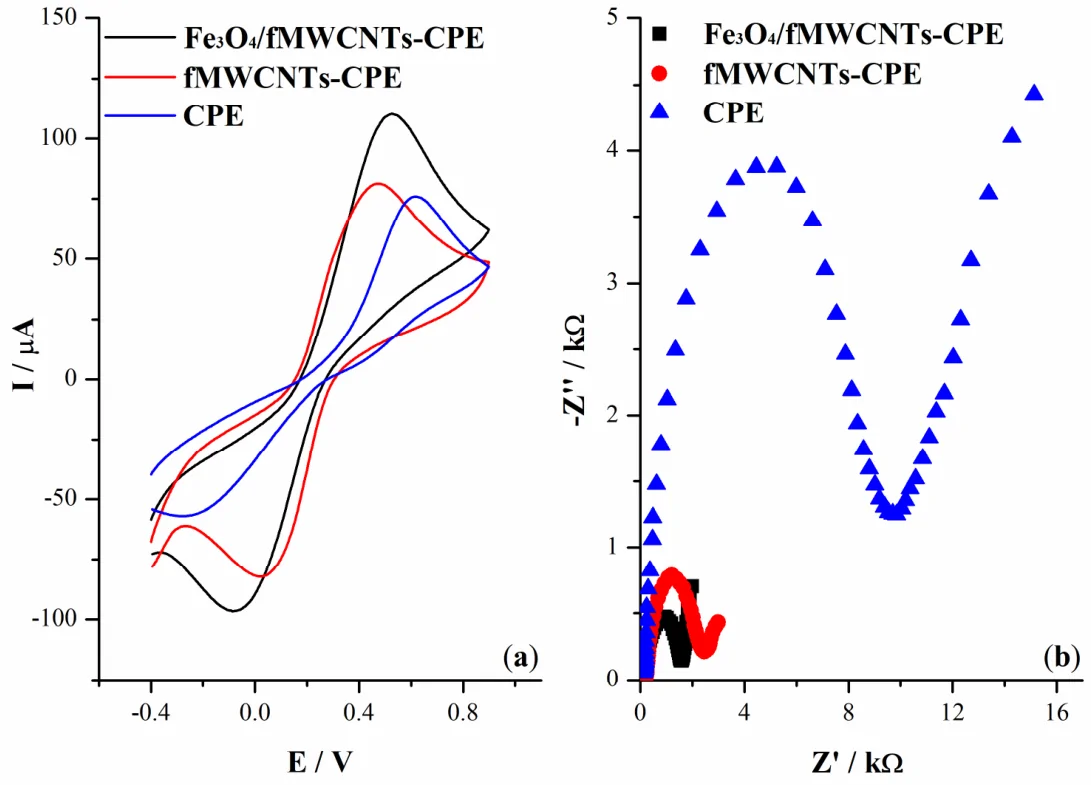
Comparative electrochemical study: (**a**) CV (scan rate of 0.1 V/s) and (**b**) Nyquist plots (the frequency ranged from 0.1 to 100,000 Hz, potential 0.2 V, amplitude 10 mV) of bare CPE, fMWCNTs-CPE, and Fe_3_O_4_/fMWCNTs-CPE in 5 mM [Fe(CN)_6_]^3−^/[Fe(CN)_6_]^4−^ in 0.1 M KCl solution.

To assess the electron-transfer process of bare CPE, fMWCNTs-CPE and Fe_3_O_4_/fMWCNTs-CPE, CV and EIS experiments were conducted using a 5 mM [Fe(CN)_6_]^3−^/[Fe(CN)_6_]^4−^ redox probe in 0.1 M KCl (Figure 5). Figure 5a shows cyclic voltammograms for all three electrodes. While modification with fMWCNTs resulted in negligible improvement, the incorporation of Fe_3_O_4_/fMWCNTs more significantly increased peak current and peak-to-peak potential separation.

The Randles–Ševčik equation was used to calculate the electroactive surface area of bare and modified CPE [42]:

*I*_p_ = 2.69 · 10^−5^ *n*^3/2^ *A C D*^1/2^ *ν*^1/2^, (1)

where *n* is the number of electrons, *A* is the electrode area (cm^2^), *C* is the concentration (mol/cm^3^), *D* is the diffusion coefficient (cm^2^/s), and *ν* is the scan rate (V/s). For Fe(CN)_6_^3−^ in 0.1 M KCl *n* = 1 and *D* = 7.6 · 10^−6^ cm^2^/s [43]. The obtained *I*_p_ values for CPE, fMWCNTs, and Fe_3_O_4_/fMWCNTs-CPE were as follows: 55.8731, 64.9091, and 97.9178 µA, while *ν* was 0.05 V/s. Using these values, the electroactive surface areas were calculated as 0.067 cm^2^ for CPE, 0.078 cm^2^ for fMWCNTs, and 0.118 cm^2^ for Fe_3_O_4_/fMWCNTs-CPE. These results indicate that the modifier not only enhances electron transfer but also increases the effective electrode surface area.

EIS was employed to gain further insights into the sensor’s properties. Figure 5b shows Nyquist plots for bare and modified CPEs, displaying a semicircle at high frequencies (indicative of charge-transfer resistance, *R_ct_*) and a straight line at low frequencies (representing diffusion). The EIS spectra were fitted using the standard Randles equivalent circuit *R_s_*-[*C* II (*R_ct_*-*W*)], consisting of *R_s_* for solution resistance, *C* for double-layer capacitance, *R_ct_* for charge-transfer resistance, and *W* for the Warburg diffusion element (Figure S2). The calculated *R_ct_* values were 8139 Ω, 1785 Ω, and 1117 Ω for bare CPE, fMWCNTs-CPE, and Fe_3_O_4_/fMWCNTs-CPE, respectively. The remaining EIS fitting parameters and chi-squared values are provided in Table S1, while the experimental and fitted EIS spectra are presented in Figure S3. The incorporation of fMWCNTs significantly reduced *R_ct_*, indicating enhanced electron transfer due to increased surface area and conductive pathways. A further reduction in *R_ct_* was observed with Fe_3_O_4_/fMWCNT modification, potentially associated with the contribution of magnetite to the interfacial properties of the modified electrode. Overall, the EIS analysis confirms that modifying the CPE with fMWCNTs and Fe_3_O_4_/fMWCNTs improves electron-transfer kinetics, thereby enhancing the sensitivity and efficiency of the electrochemical sensor.

### 3.6. Electrochemical Oxidation Behavior of EST

The composition and pH of the supporting electrolyte significantly influence key voltammetric parameters, including peak height, shape, width, and position. In this study, the voltammetric behavior of EST was examined in various supporting electrolytes, including acetate, citrate, Britton–Robinson, and phosphate buffers. A single oxidation peak corresponding to EST was observed in all tested supporting electrolytes. Acidic conditions enhanced EST oxidation, particularly improving peak height and sharpness, whereas higher pH resulted in lower intensity and broader peaks. Phosphate buffer yielded the highest analytical signal, confirming it as the most suitable medium for EST determination.

**Scheme 1:**
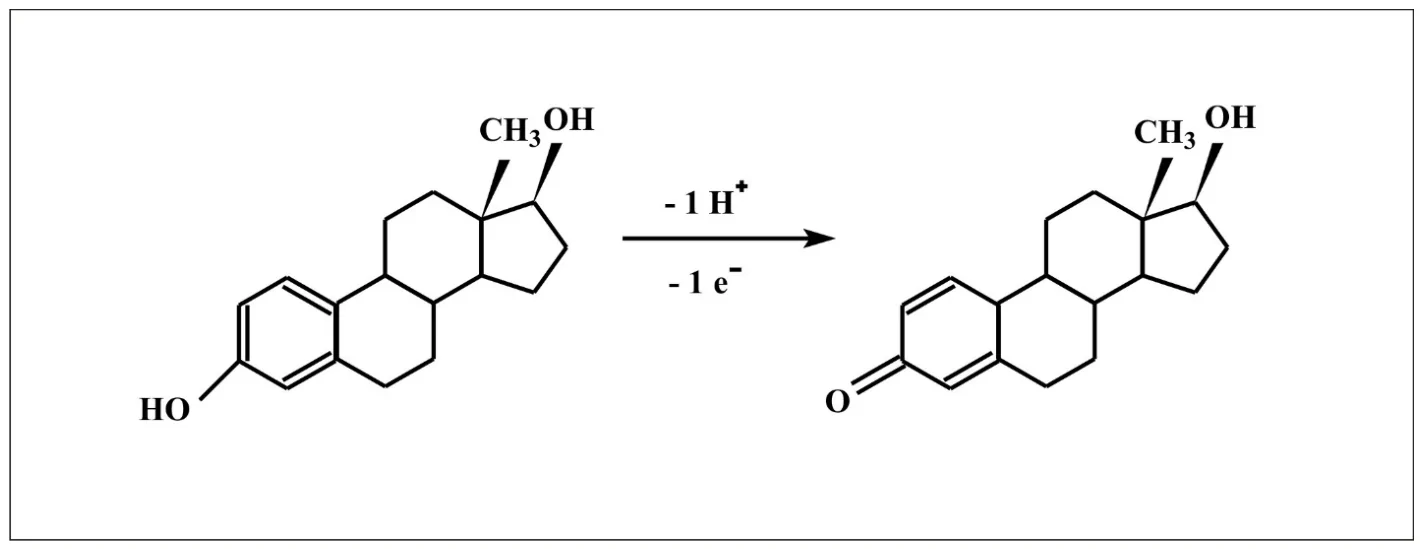
The proposed oxidation mechanism of EST.

The influence of pH on the oxidation peak current and potential in phosphate buffer was subsequently examined. CV and SWV were performed in 0.1 M phosphate buffer containing 20 µM EST over a pH range of 3.0 to 8.0. The peak current increased with increasing pH, reaching a maximum at pH 5 before decreasing at higher pH values. Additionally, the oxidation potential shifted towards less positive values as the pH increased, indicating proton involvement in the oxidation process (Figure S4). This dependence was linear and was described by the equation *E*_p_ = −0.05 pH + 1.05 (R^2^ = 1), with a slope of −0.05 V/pH, suggesting that an equal number of protons and electrons are involved in the redox process. Although this relationship does not establish the absolute number of electrons transferred, previous studies [34,36,44,45] have proposed an oxidation mechanism involving the transfer of one proton and one electron through the oxidation of the hydroxyl group on the aromatic ring, resulting in the formation of the corresponding ketone derivative (Scheme 1). The mechanism proposed herein is therefore based on the present pH-dependent electrochemical response in conjunction with these previously reported findings.

To further investigate the electrochemical oxidation behavior of EST at the Fe_3_O_4_/fMWCNTs-CPE, the effect of scan rate on the oxidation peak current was studied. CVs of 5 µM EST were recorded in 0.1 M phosphate buffer (pH 5.0) at scan rates ranging from 0.05 to 0.5 V/s (Figure S5). No corresponding cathodic peak was observed on the reverse scan at the investigated scan rates (Figure S5a), suggesting that the oxidation process is electrochemically irreversible under the applied experimental conditions, which is consistent with previous studies [34,36,37,44,45,46,47]. The anodic peak current increased with increasing scan rates. A linear relationship was observed between the anodic peak current and the square root of the scan rate (R^2^ = 0.9935, Figure S5b), described by the equation *I*_p_ = 0.0134 *ν*^0.5^ − 0.0012 (µA, (V/s)^1/2^), indicating a predominantly diffusion-controlled oxidation process. Additionally, the plot of log *I*_p_ versus log ν (Figure S5c) exhibited a linear trend with a slope close to the theoretical value of 0.5, further supporting the predominance of diffusion-controlled transport. Nevertheless, the shift in oxidation peak potential with increasing scan rate suggests that electron-transfer kinetics may also influence the electrochemical response.

### 3.7. Analytical Determination of EST

#### 3.7.1. Optimization of the Voltammetric Parameters

To develop a rapid and sensitive SWV method for detecting EST in various samples, the influence of key electrochemical parameters—pulse amplitude (*E*_sw_), frequency (*f*), step potential (*ΔE*), and equilibration time (*t*_eq_)—on the EST oxidation response was investigated. Experiments were conducted with 5 µM EST in 0.1 M phosphate buffer (pH 5.0). The effect of pulse amplitude was examined within the 10–200 mV range. The anodic peak current increased from 10 to 50 mV, slightly decreased between 50 and 100 mV, and dropped significantly beyond 100 mV, with peak broadening observed above 50 mV (Figure S6a). Frequency was varied from 1 Hz to 100 Hz, showing an increase in peak current up to 50 Hz, followed by signal distortion and a slight decrease beyond this point (Figure S6b). Step potential was tested within 1–20 mV, with an increase in the analytical signal up to 7.5 mV; beyond this value, signal distortion and reduced reproducibility were observed (Figure S6c). Finally, the equilibrium time was varied between 0 and 20 s. While a slightly lower signal was observed at 0 s, it provided optimal reproducibility, whereas longer equilibration times (5–20 s) led to baseline curvature and diminished reproducibility (Figure S6d). Based on these findings, the optimal SWV parameters were determined as 50 mV for *E*_sw_, 50 Hz for *f*, 7.5 mV for *ΔE*, and 0 s for *t*_eq_.

#### 3.7.2. Analytical Figures of Merit

**Figure 6:**
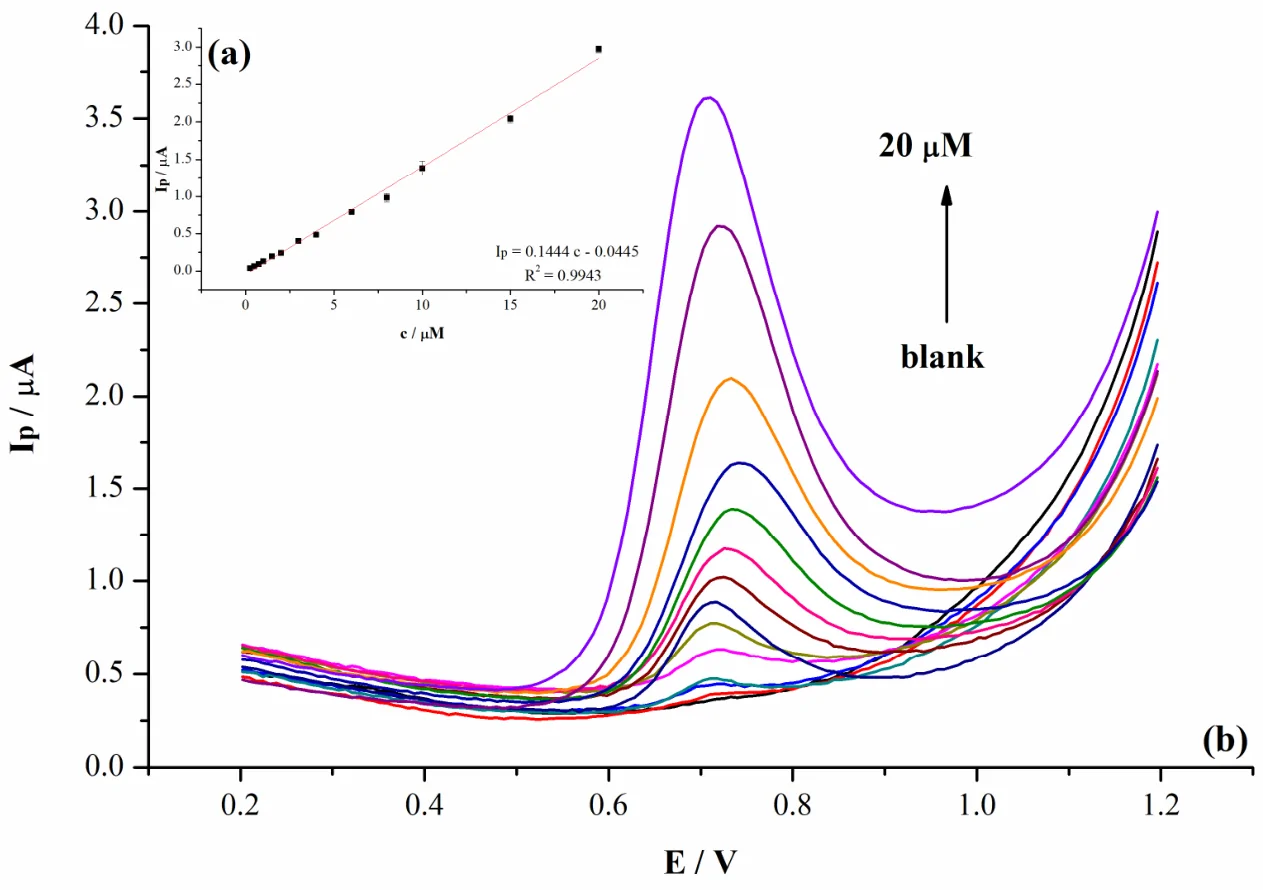
(**a**) Calibration curve and (**b**) SWVs for EST detection using Fe_3_O_4_/fMWCNTs-CPE. SWVs were recorded for blank and EST concentrations of 0.25, 0.5, 0.75, 1, 1.5, 2, 3, 4, 6, 8, 10, 15, and 20 µM (*E*_sw_ = 50 mV, *f* = 50 Hz, *ΔE* = 7.5 mV, *t*_eq_ = 0 s). Error bars on the calibration curve represent the standard deviations of five replicate measurements (*n* = 5).

Following the optimized parameters outlined in previous sections, the linearity of the method was assessed by constructing a calibration curve of peak current (*I*_p_) versus EST concentration. A linear dynamic range of 0.25–20 µM was achieved, as illustrated in Figure 6, with the relationship expressed as *I*_p_ (µA) = 0.1444 *c* (µM)–0.04454 (R^2^ = 0.9943). The statistical confidence intervals for the slope and intercept of the calibration curve are provided in Table S2. The limit of detection (LOD) and limit of quantification (LOQ) were calculated using the equations LOD = 3.3 *σ*/*S* and LOQ = 10 *σ*/*S*, where *σ* represents the standard deviation of the intercept obtained from five independently constructed calibration curves, and *S* denotes the mean slope of the calibration curve [48]. The LOD and LOQ were determined to be 0.0215 µM and 0.0651 µM, respectively.

The method’s precision was assessed regarding repeatability (intra-day precision) and reproducibility (inter-day precision) by conducting ten replicate measurements (*n* = 10) on a standard EST solution (2 µM). Intra-day measurements were conducted within a single day, while inter-day measurements were performed over ten consecutive days using the same batch of prepared carbon paste. During the measurements, the electrode holder was replenished with the remaining paste from the same batch when necessary. The results, shown in Figure S7, indicate that the relative standard deviation (RSD) values remained below 3.7% for both intra- and inter-day assays, demonstrating the high precision of the proposed method. Reproducibility was further assessed using nine independently prepared carbon pastes following the same preparation procedure. The RSD obtained from measurements performed using electrodes prepared from these pastes was 4.28%. Between experimental sessions, the carbon paste was stored in a closed cuvette or in a carbon paste holder in a refrigerator. Detailed analytical characteristics are provided in Table 1.

Table 2 presents a comparison of the analytical performance of the developed method with other voltammetric techniques for EST determination, emphasizing parameters such as linear range and detection limit using modified CPEs. Notably, a graphene-ordered mesoporous carbon composite (GR/OMC/CPE) used as a CPE modifier has achieved an exceptionally low detection limit (0.002 µM) [37]. Another significant study employing CPE/GNR-FS-Au-CA (cysteamine self-assembled gold nanoparticle-modified fumed silica decorated graphene nanoribbon nanocomposite) demonstrated a remarkable detection limit of 0.0074 µM and extended applicability to pharmaceutical formulations and milk, with a linear range reaching 5 µM [36].

Similar detection limits have been obtained for CPEs modified with copper (II) oxide (CuO/CPE) and nitric acid-treated fumed silica (NT-FS/CPE), with practical application in food, urine, and pharmaceutical samples [34,35]. Magnetite has previously been used as a modifier due to its large surface area, excellent conductivity, and magnetic properties, which facilitate electrode modification and improve electrochemical response. In combination with an ionic liquid, it has been applied for EST determination in pharmaceutical formulations [38]. However, despite these advantages, this approach resulted in a limited linear range and a higher detection limit. Magnetite has also been incorporated into magnetic molecularly imprinted polymer (MagMIP) for the determination of EST in water samples [39]. Although this approach provided enhanced selectivity due to modification, the sensor exhibited moderate analytical performance, with a detection limit higher than those reported for several other magnetite-based EST sensors. The sensor developed in this study exhibits a slightly narrower linear range than certain reported methodologies; its broader applicability to real samples, combined with the advantages of the chosen modification strategy, enhances its practical utility.

#### 3.7.3. Interferences

The selectivity of the developed method was evaluated by testing potential interferents commonly found in water and urine samples. Standard solutions of possible interfering agents, including inorganic ions (Na^+^, K^+^, PO_4_^3−^, SO_4_^2−^, NH_4_^+^, and SO_3_^2−^) and organic substances (ascorbic acid, urea, creatinine, cysteine, and uric acid), were individually added to a solution containing 2 µM of EST. The interference was assessed by monitoring the anodic peak current using SWV, with a 200-fold excess of each substance. The tolerance limit was defined as the maximum concentration of the interfering substance that caused less than a 5% change in the signal. The results indicated that none of the tested substances, except uric acid, caused significant interference, with variations in the analytical signal remaining below 5% (Figure S8). Uric acid did not interfere at a 40-fold excess; at a 50-fold excess, the interference was 5.06%, while higher concentrations led to noticeable interference. These findings confirm the high selectivity of the method for EST, supporting its reliable application in complex matrices such as urine and water.

#### 3.7.4. Analysis of the Real Samples

**Figure 7:**
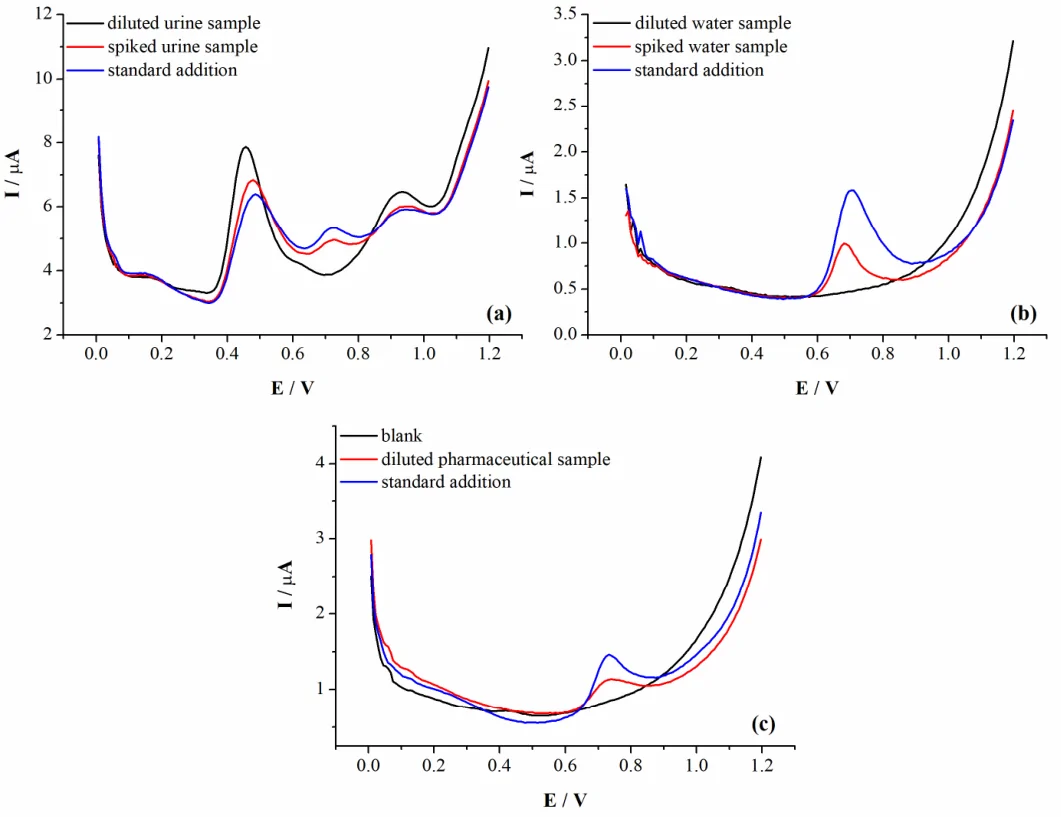
SWVs for EST determination using Fe_3_O_4_/fMWCNTs-CPE in phosphate buffer (pH = 5.0) with the standard addition method in real samples: (**a**) urine, (**b**) water, and (**c**) pharmaceutical preparation. The EST concentration (spiked or analyzed) was 2.5 μM. (*E*_sw_ = 50 mV, *f* = 50 Hz, *ΔE* = 7.5 mV, *t*_eq_ = 0 s).

To evaluate the practical applicability of the developed sensor, Fe_3_O_4_/fMWCNTs-CPE was employed for the determination of EST in human urine, water samples, and EST tablets. Sample preparation followed previously described procedures, while quantification was performed using the standard addition method. SW voltammograms for urine, water, and pharmaceutical samples are shown in Figure 7. No detectable EST signal was observed in the diluted urine and tap-water samples under the applied experimental conditions; therefore, these samples were spiked with known amounts of EST. The results, summarized in Table 3 (triplicate analysis), show hormone recoveries calculated as the ratio of determined EST concentration to the corresponding expected concentration, expressed as a percentage. The obtained recovery values ranged between 98.53% and 101.79%, with relative standard deviations (RSD) below 2.40%. These results provide supporting evidence for the accuracy and precision of the proposed method. Despite the potential interference of uric acid indicated by the selectivity study, the EST oxidation peak remained identifiable in the voltammogram of the urine sample (Figure 7). The satisfactory recovery obtained for the diluted, spiked urine sample further supports the applicability of the proposed method to the analyzed sample. The findings provide a preliminary indication of the potential applicability of the modified CPE sensor for EST determination in urine, water, and pharmaceutical products.

## 4. Conclusions

In this study, an Fe_3_O_4_/fMWCNTs-modified carbon paste electrode (Fe_3_O_4_/fMWCNTs-CPE) was successfully developed and applied for the electrochemical determination of EST using SWV. The Fe_3_O_4_/fMWCNT composite was synthesized via the wet impregnation method, and FE-SEM and EDX characterization confirmed the uniform distribution of Fe_3_O_4_ nanoparticles on the fMWCNTs. Incorporating this composite as a modifier in the carbon paste enhanced EST oxidation by increasing the electrode surface area and improving electron-transfer kinetics.

The developed SWV method exhibited a reliable linear range, with a relatively low detection limit compared to the literature, demonstrating its suitability for sensitive EST determination. Precision studies showed low RSD values for intra- and inter-day measurements, confirming the method’s reproducibility. Selectivity tests indicated that most of the investigated substances caused minor signal variations, remaining below 5% even at a 200-fold excess, whereas uric acid interfered at higher concentrations. The sensor was applied to EST determination in one urine sample, one water sample, and one sample of pharmaceutical tablets, with high recoveries. These findings indicate satisfactory recovery and precision under the investigated conditions and provide a preliminary indication of the potential applicability of Fe_3_O_4_/fMWCNTs-CPE combined with SWV for EST determination in these matrices.

## Figures and Tables

**Table 1:** Analytical parameters for EST detection using SWV with Fe_3_O_4_/fMWCNTs-CPE in 0.1 M phosphate buffer (pH 5.0).

Linear concentration range (µM)	0.25–20
Slope (µA/µmol) ^a^	0.1444
Intercept (µA) ^a^	−0.0445
Correlation coefficient (R^2^) ^a^	0.9943
RSD slope (%) ^a^	1.0608
RSD intercept (%) ^a^	2.1097
LOD (µM)	0.0215
LOQ (µM)	0.0651
Repeatability (intra-day, RSD, %) ^b^	2.9975
Reproducibility (inter-day, RSD, %) ^b^	3.6220
Reproducibility (independently prepared sensors, RSD, %) ^c^	4.2796

^a^*n* = 5; ^b^ *n* = 10; ^c^ *n* = 9.

**Table 2:** Comparison of electroanalytical methods found in the literature for EST determination using modified CPEs.

Electrode/Modifier	Method	Linearity Range (µM)	LOD (µM)	Analyzed Sample	Reference
GR/OMC/CPE	SWV	0.005–2 (5.0 × 10^−9^–2 × 10^−6^ M)	0.002 (2.0 × 10^−9^ M)	blood serum	[37]
CPE/GNR-FS-Au-CA	DPV	0.1–5.0	0.0074	milk and pharmaceutical sample	[36]
CuO/CPE	SWV	0.06–0.08 (60.0–80.0 nM)	0.021 (21.0 nM)	urine and buttermilk samples	[34]
NT-FS/CPE	DPV	0.1–15.0	0.023	milk and pharmaceutical samples	[35]
Fe_3_O_4_NPs-BMI.PF_6_/CPE	SWV	0.1–1	0.05 (50 nM)	pharmaceutical formulations	[38]
CPE-MagMIP	DPAdSV	0.5–14.0	0.13	water samples	[39]
Fe_3_O_4_/fMWCNTs-CPE	SWV	0.25–20	0.022	water, urine samples, pharmaceutical formulation	This work

GR/OMC/CPE—electrodeposited graphene ordered mesoporous carbon composite-modified carbon paste electrode; CPE/GNR-FS-Au-CA—cysteamine self-assembled gold nanoparticle modified fumed silica decorated graphene nanoribbon nanocomposite-modified CPE; CuO/CPE—copper (II) oxide-modified CPE; NT-FS/CPE—nitric acid-treated fumed silica-modified CPE; Fe_3_O_4_NPs-BMI.PF_6_/CPE—magnetite nanoparticles and ionic liquid 1-butyl-3-methylimidazolium hexafluorophosphate-modified CPE; CPE-MagMIP—magnetic molecularly imprinted polymer; Fe_3_O_4_/fMWCNTs-CPE—carbon paste electrode modified with Fe_3_O_4_ functionalized MWCNT.

**Table 3:** Results for EST determination in urine, water samples, and pharmaceutical formulation using the proposed electroanalytical method with Fe_3_O_4_/fMWCNTs-CPE.

Sample	Added (µmol/L)	Found ^a^ (µmol/L)	Recovery ^b^ (%)	RSD (%)
urine	2.50	2.52	100.69	1.30
	5.00	5.04	100.90	1.96
water	1.25	1.24	99.30	2.34
	2.50	2.54	101.59	1.03
	4.50	4.58	101.79	0.97
tablet	2.50	2.46	98.53	2.08

^a^*n* = 3, ^b^ Recovery = Found/spiked × 100.

## Data Availability

Data will be made available on demand.

## Supplementary Materials

The following supporting information can be downloaded at: https://www.mdpi.com/article/10.3390/s26216917/s1, Figure S1: Exploring the impact of varied Fe_3_O_4_/fMWCNTs content in CPE on anodic peak current (*I_p_*) in EST sensing: (a) SWVs; (b) Anodic peak current. C_EST_ = 20 μM in PBS (pH 5.0); Figure S2: Equivalent electrical circuit used for EIS data fitting; Table S1: EIS fitting parameters for bare CPE, fMWCNTs-CPE, and Fe_3_O_4_/fMWCNTs-CPE; Figure S3: Experimental and fitted Nyquist plots for bare CPE, fMWCNTs-CPE, and Fe_3_O_4_/fMWCNTs-CPE; Figure S4: The effect of phosphate buffer pH on the peak current and anodic peak potential. CVs are recorded in 0.1 M phosphate buffer containing 20 µM of EST (scan rate 0.3 V/s, *ΔE* = 0.05 V) using Fe_3_O_4_/fMWCNTs-CPE; Figure S5: (a) Cyclic voltammograms recorded at different scan rates; (b) Dependence of the peak current (*I*_p_) on the square root of the scan rate (*ν*^0.5^); (c) Logarithmic plot of peak current (log *I*_p_) versus scan rate (log *ν*) for CV on Fe_3_O_4_/fMWCNTs-CPE in 5 µM EST in 0.1 M phosphate buffer (pH 5) (scan rate from 0.05 to 0.5 V/s, *ΔE* = 50 mV); Figure S6. Optimization of SWV parameters for 5 µM EST at Fe_3_O_4_/fMWCNTs-CPE in phosphate buffer (pH 5.0) (a) Effect of *E*_sw_ *(f* = 50 Hz, *ΔE* = 5 mV, *t*_eq_ = 5 s); (b) Effect of *f* (*E*_sw_ = 50 mV, *ΔE* = 5 mV, *t*_eq_ = 5 s); (c) Effect of *ΔE* (*E*_sw_ = 50 mV, *f* = 50 Hz, *t*_eq_ = 5 s); (d) Effect of *t_eq_* (*E*_sw_ = 50 mV, *f* = 50 Hz, *ΔE* = 7.5 mV); Table S2. Linear regression parameters and 95% confidence intervals for the calibration curve; Figure S7. Precision of the method: SWVs for (a) Intra-day repeatability; (b) Inter-day reproducibility for the analysis of 2 µM EST using Fe_3_O_4_/fMWCNTs-CPE in 0.1 M phosphate buffer (pH 5.0); Figure S8. Relative variation of peak current in the presence of different interferents (200-fold excess relative to EST concentration) during SW voltammetric determination of EST with Fe_3_O_4_/fMWCNTs-CPE in phosphate buffer (pH = 5.0) (*E*_sw_ = 50 mV, *f* = 50 Hz, *ΔE* = 7.5 mV, *t*_eq_ = 0 s).

## Abbreviations

The following abbreviations are used in this manuscript.

EST	Estradiol
CPE	Carbon paste electrode
fMWCNTs	Functionalized multi-walled carbon nanotubes
fMWCNTs-CPE	CPE modified with fMWCNTs
Fe_3_O_4_/fMWCNTs-CPE	CPE modified with magnetite fMWCNTs
FE-SEM	Field emission–scanning electron microscopy
EDX	Energy-dispersive X-ray spectroscopy
XRD	X-ray powder diffraction analysis
CV	Cyclic voltammetry
EIS	Electrochemical impedance spectroscopy
SWV	Square-wave voltammetry
R_ct_	Charge-transfer resistance
E_sw_	Pulse amplitude
*f*	Frequency
ΔE	Step potential
t_eq_	Equilibration time
LOD	Limit of detection
LOQ	Limit of quantification
RSD	Relative standard deviation
