# Supplementary material for: Electrochemical Sensing of Estradiol Using an Fe3O4/fMWCNT-Modified Carbon Paste Electrode
Source: Sensors (Basel). 2026 Oct 31;26(21):6917. doi: 10.3390/s26216917 (PMC13766390; doi:10.3390/s26216917)
Supplement: Supplementary file 1 [file sensors-26-06917-s001.zip › sensors-4617933-supplementary.pdf]

Electrochemical sensing of estradiol using an Fe_3O_4/fMWCNT-modified carbon paste electrode

Ana Đurović[1,*], Zorica Stojanović[1], Sanja Panić[1], Marija Milanović[1], Snežana Kravić[1], Lukáš Richtera[2]

[1] University of Novi Sad, Faculty of Technology Novi Sad, Bul. cara Lazara 1, Novi Sad, Serbia

[2] Department of Chemistry and Biochemistry, Mendel University in Brno, Brno, Zemědělská 1, 613 00 Brno, Czech Republic

[*]Correspondence: ana.djurovic@uns.ac.rs; Tel.: +381-21-485-3658

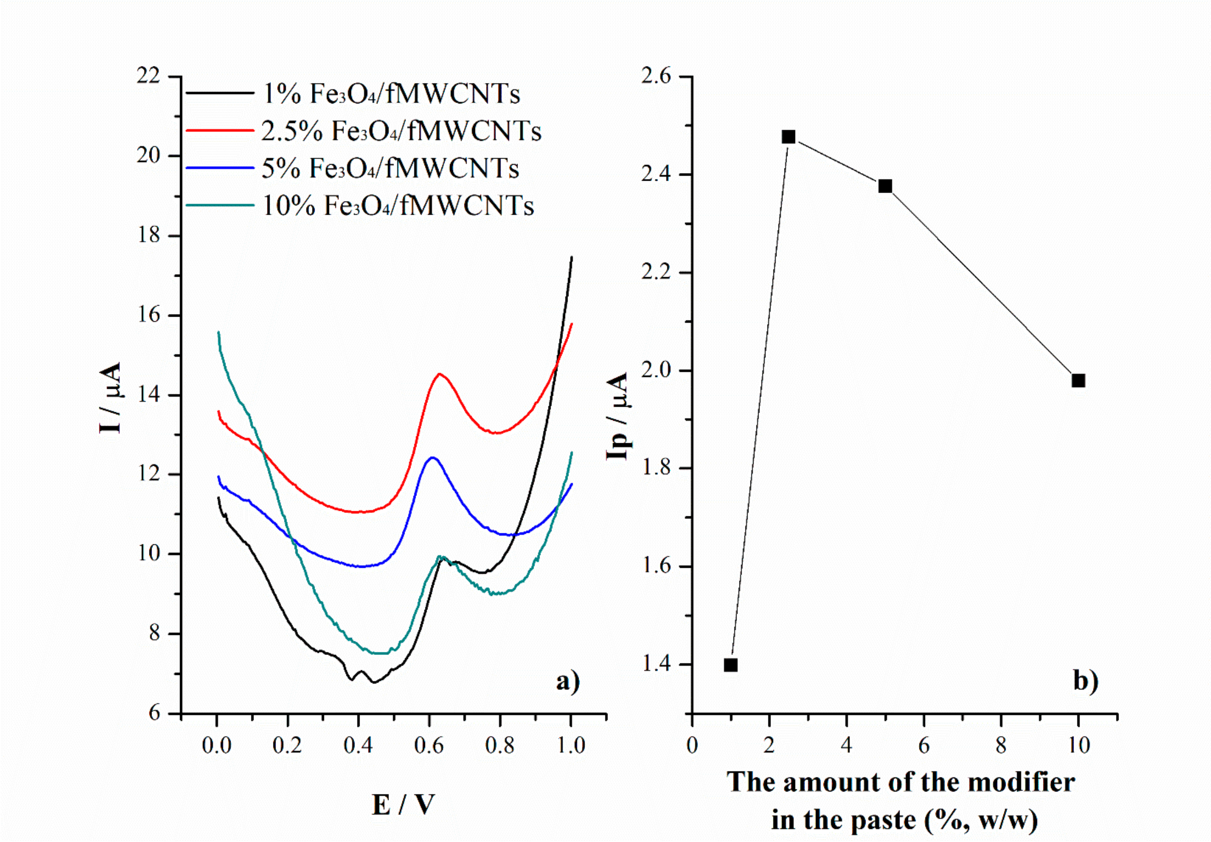

Figure S1. Exploring the impact of varied Fe_3O_4/fMWCNT content in CPE on anodic peak current (Ip) in EST sensing: a) SWVs; b) anodic peak current. C_{EST} = 20 μM in PBS (pH 5.0).

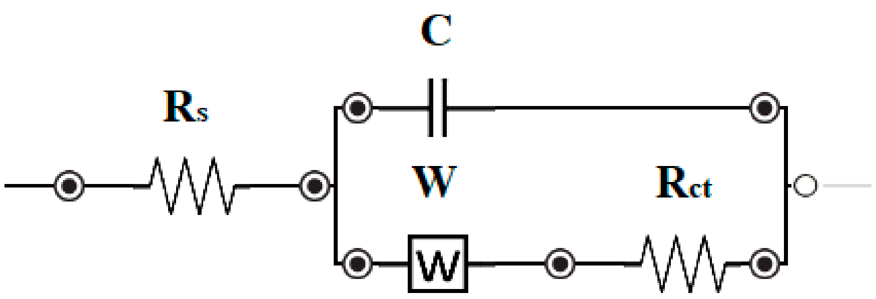

Figure S2. Equivalent electrical circuit used for EIS data fitting.

Table S1. EIS fitting parameters for bare CPE, fMWCNTs-CPE, and Fe_3O_4/fMWCNTs-CPE.

Working electrode	R_s (Ω)	R_{ct} (Ω)	C (nF)	W parameter (kσ)	χ^2
CPE	148.8	8139	148.9	4.576	0.0286
fMWCNTs-CPE	222.1	1785	1264	0.635	0.0308
Fe_3O_4/fMWCNTs-CPE	174.1	1117	632.6	0.593	0.0231

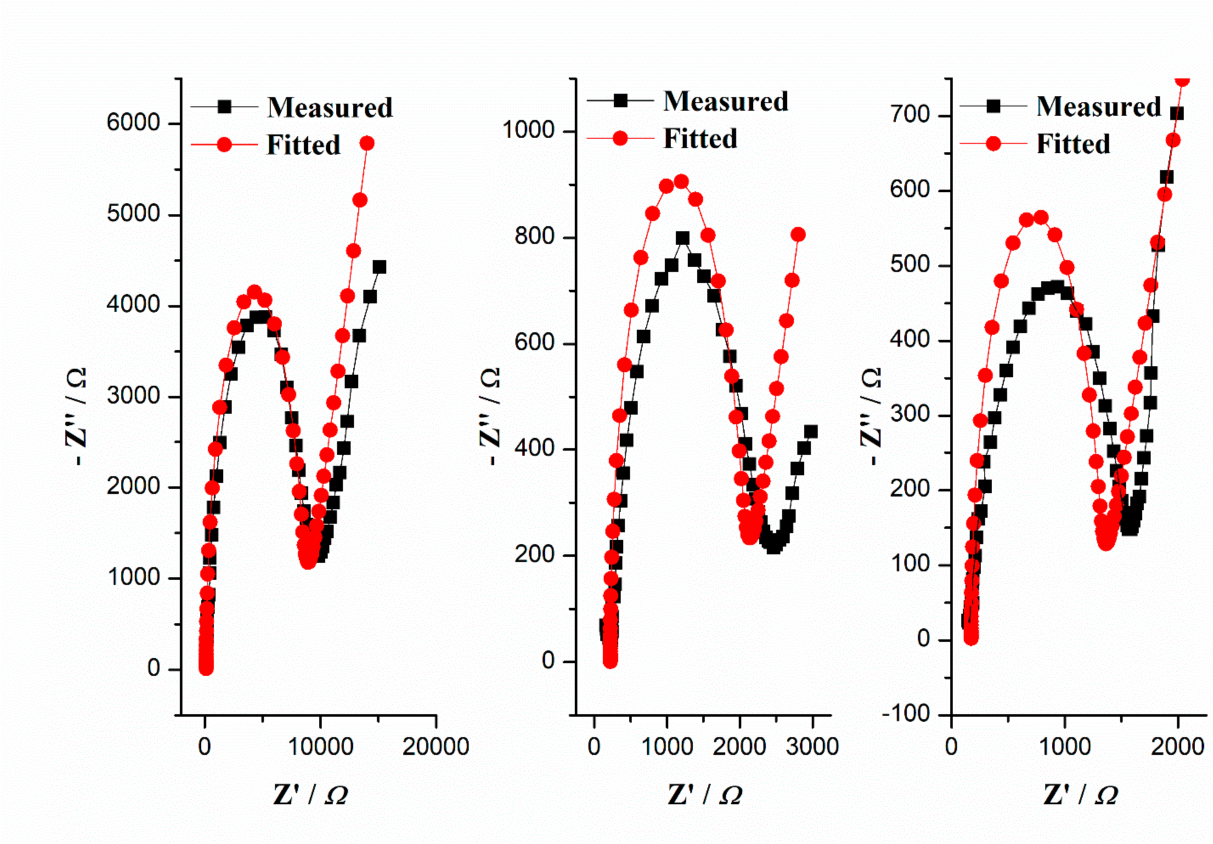

Figure S3. Experimental and fitted Nyquist plots for bare CPE, fMWCNTs-CPE, and Fe_3O_4/fMWCNTs-CPE.

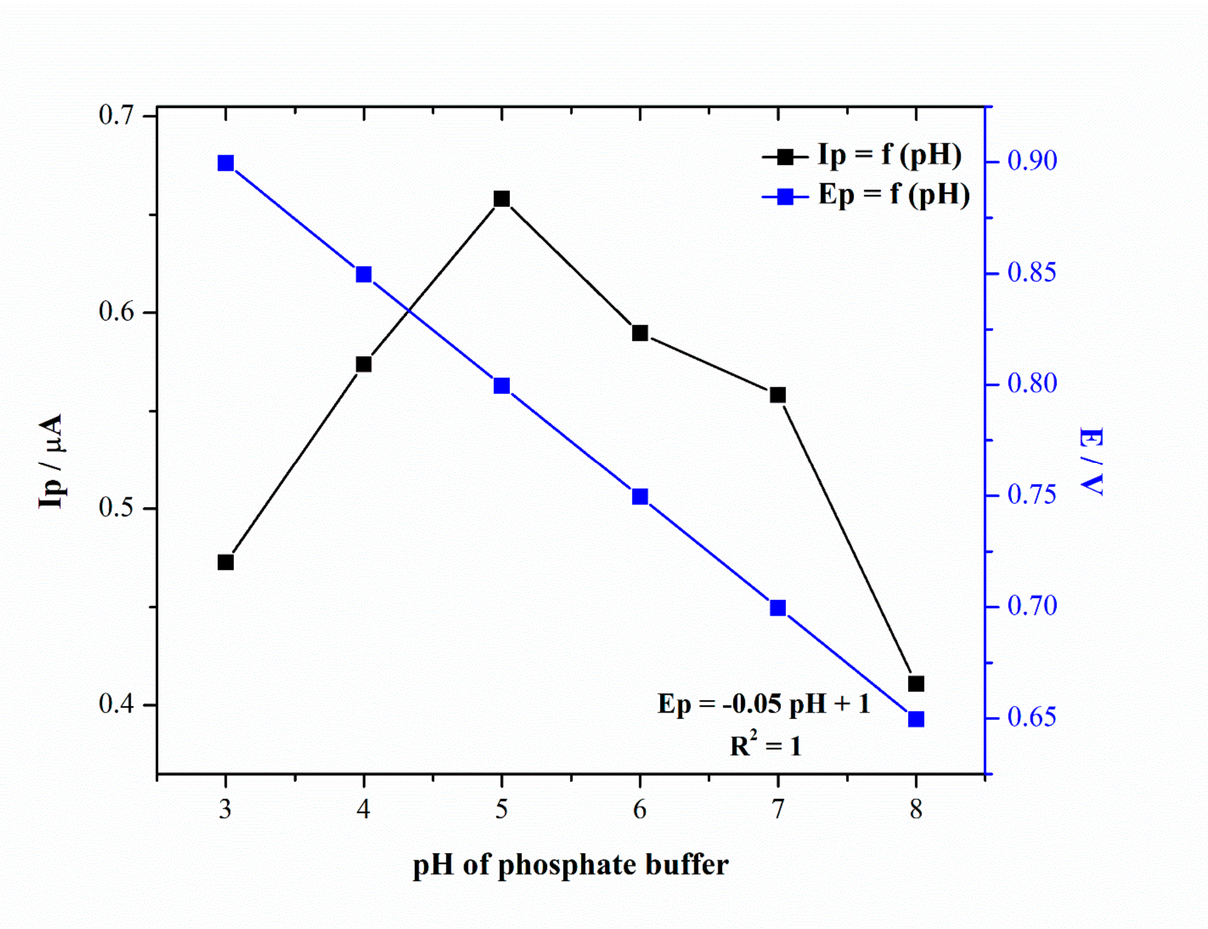

Figure S4. The effect of phosphate buffer pH on the peak current and anodic peak potential. CVs were recorded in 0.1 M phosphate buffer containing 20 µM of EST (scan rate 0.3 V/s, Estep = 0.05 V) using Fe_3O_4/fMWCNTs-CPE.

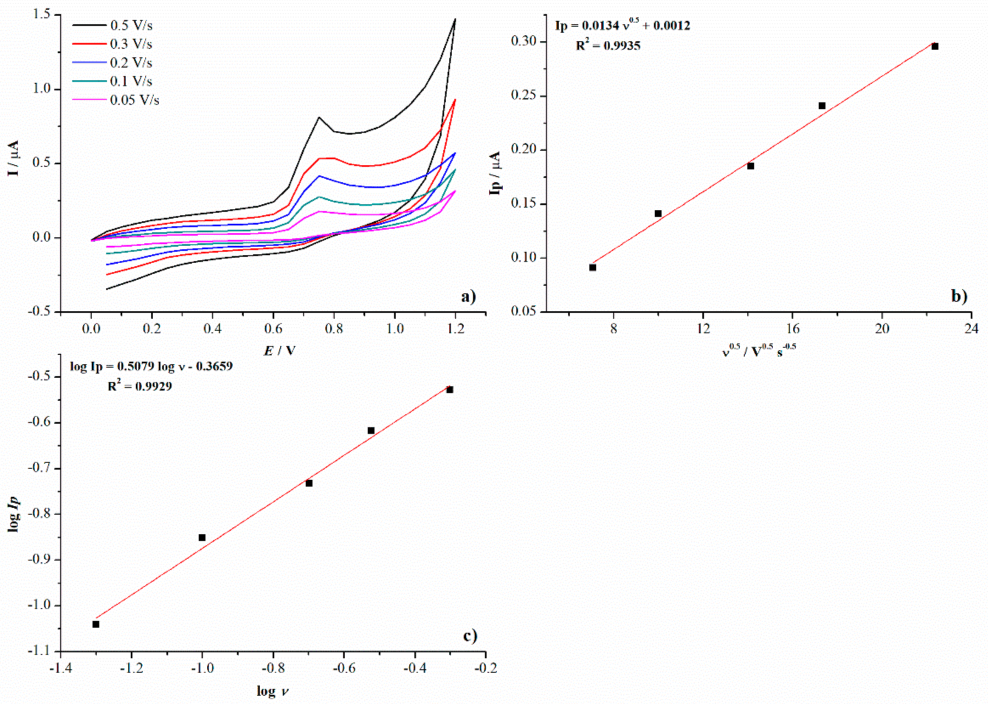

Figure S5. a) Cyclic voltammograms recorded at different scan rates. b) Dependence of the peak current (Ip) on the square root of the scan rate ($\nu^{0.5}$). c) Logarithmic plot of peak current (log Ip) versus scan rate (log ν) for CV on Fe_3O_4/fMWCNTs-CPE in 5 µM EST in 0.1 M phosphate buffer (pH 5) (scan rate from 0.05 to 0.5 V/s, ΔE = 50 mV).

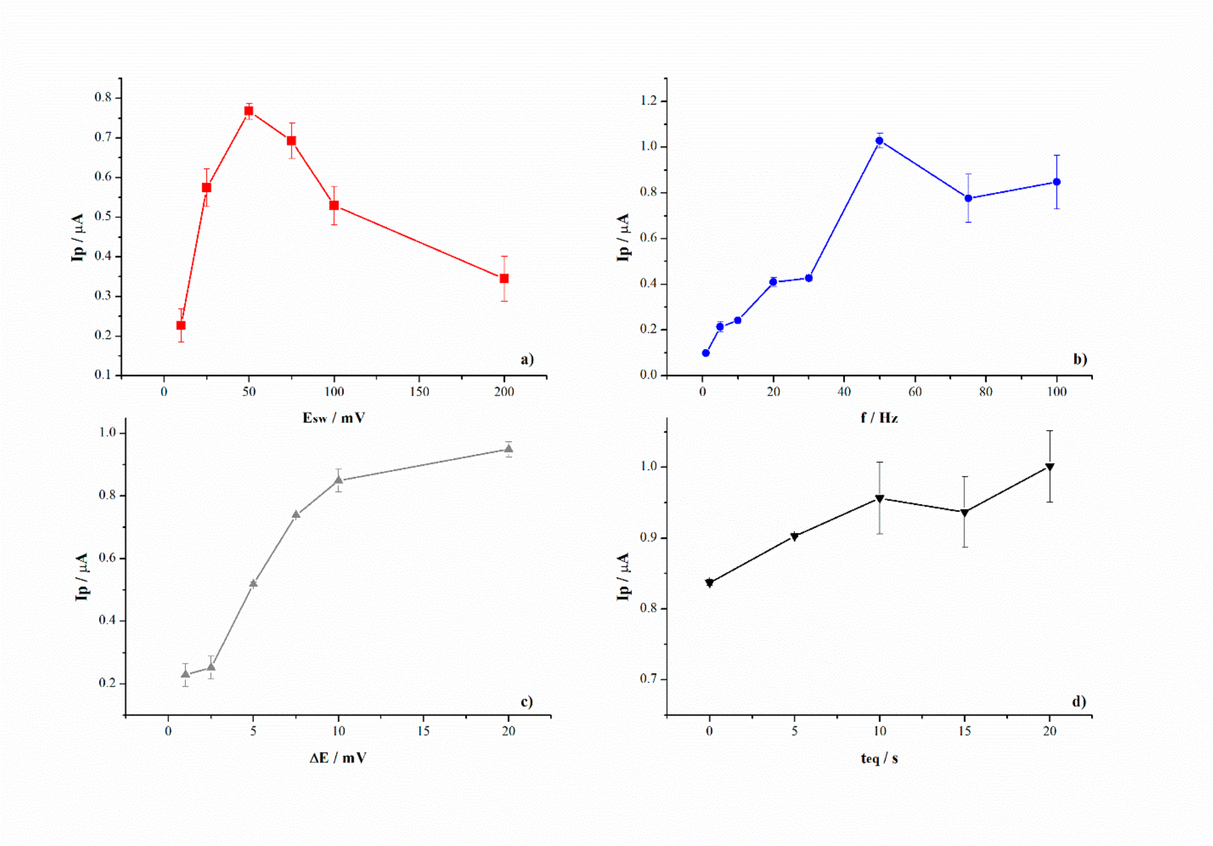

Figure S6. Optimization of SWV parameters for 5 µM EST at Fe_3O_4/fMWCNTs-CPE in phosphate buffer (pH 5.0) a) Effect of E_{sw} (f = 50 Hz, ΔE = 5 mV, t_{eq} = 5 s); b) Effect of f (E_{sw} = 50 mV, ΔE = 5 mV, t_{eq} = 5 s); c) Effect of ΔE (E_{sw} = 50 mV, f = 50 Hz, t_{eq} = 5 s); d) Effect of t_{eq} (E_{sw} = 50 mV, f = 50 Hz, ΔE = 7.5 mV).

Table S2. Linear regression parameters and 95% confidence intervals for the calibration curve.

Parameter	Estimate	95% CI (lower)	95% Cl (upper)
Slope	0.1444	0.1375	0.1513
Intercept	-0.0445	-0.1009	0.0118

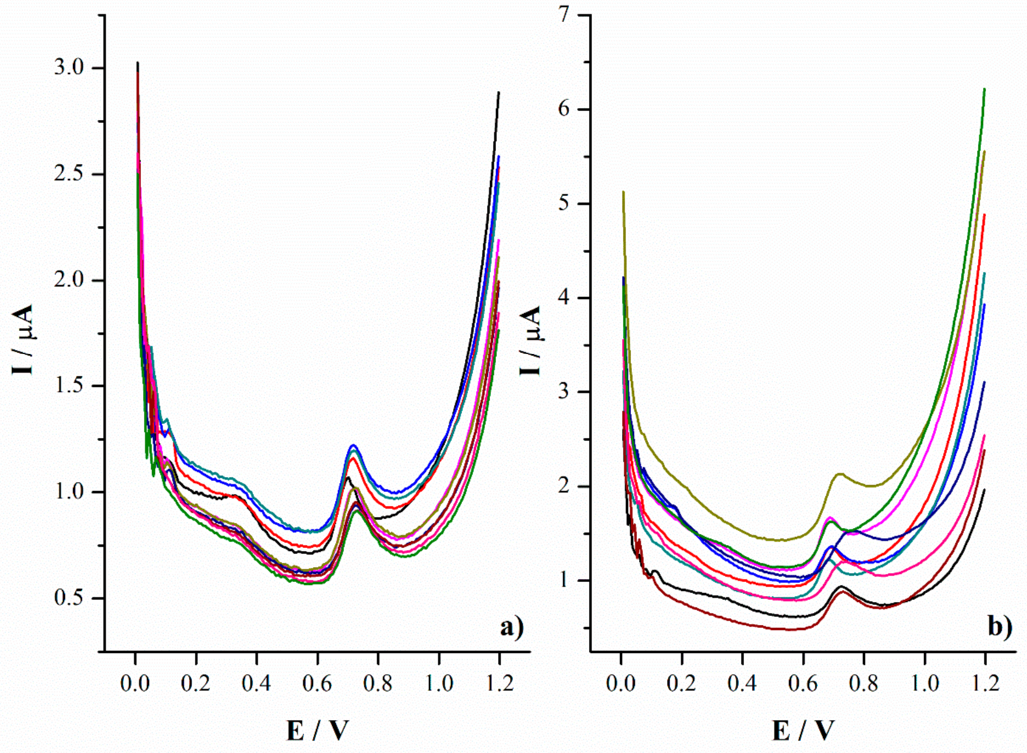

Figure S7. Precision of the method: SWVs for a) intra-day repeatability and b) inter-day reproducibility for the analysis of 2 µM EST using Fe_3O_4/fMWCNTs-CPE in 0.1 M phosphate buffer (pH 5.0).

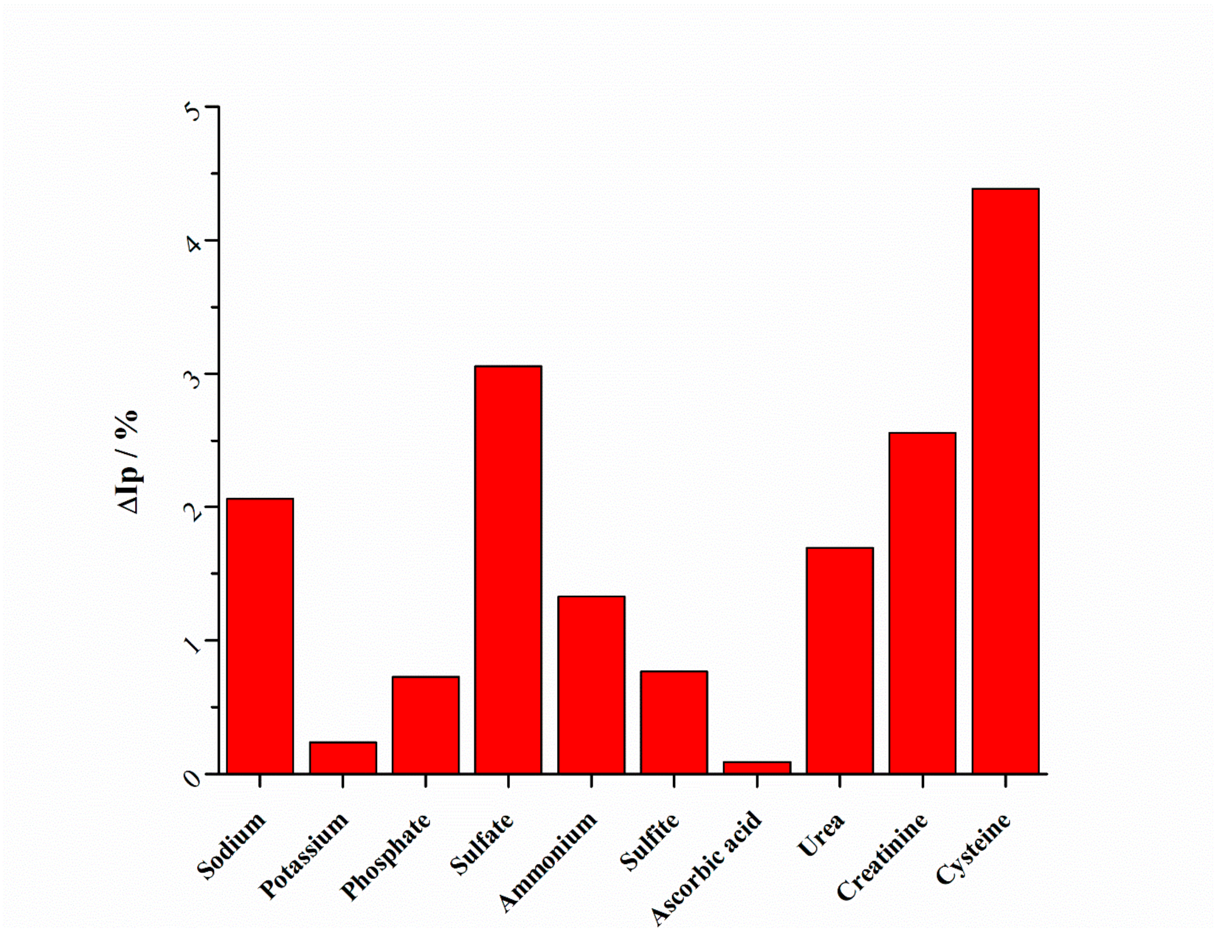

Figure S8. Relative variation in peak current in the presence of different interferents (200-fold excess relative to EST concentration) during SW voltammetric determination of EST with Fe_3O_4/fMWCNTs-CPE in phosphate buffer (pH = 5.0) (E_{sw} = 50 mV, f = 50 Hz, ΔE = 7.5 mV, t_{eq} = 0 s).
